# Health related quality of life (HRQoL) among Aboriginal South Australians: a perspective using survey-based health utility estimates

**DOI:** 10.1186/s12955-019-1107-z

**Published:** 2019-02-18

**Authors:** David Banham, Jonathan Karnon, John Lynch

**Affiliations:** 1 School of Public Health, University of Adelaide, North Terrace, Adelaide, South Australia 5000; 2 Wardliparingga Aboriginal Health Research, South Australian Health and Medical Research Institute, North Terrace, Adelaide, South Australia 5000; 3 Division of Health Sciences, University of South Australia, North Terrace, Adelaide, South Australia 5000

**Keywords:** Health related quality of life, Health utility, SF-6D, Patient reported outcome measures, Aboriginal health, Disparities, Health inequities

## Abstract

**Background:**

Australian health surveys occasionally include health utility measures in describing health related quality of life (HRQoL) across the general population. However, the HRQoL of specific population groups, such as Aboriginal and Torres Strait Islander (respectfully referred to as Aboriginal), are poorly understood. Our analysis describes HRQoL utility among Aboriginal South Australians by examining the characteristics of respondents completing HRQoL questioning, the relationship between HRQoL and respondent characteristics, then considers reported HRQoL utility in the wider population context.

**Methods:**

Population weighted and self-reported HRQoL was measured using SF-6D, as derived from the SF-12 version 2, in the South Australian Aboriginal Health Survey’s face to face interviews with 399 respondents aged 15 or more in 2010/11.

**Results:**

Mean HRQoL utility was 0.77 (95% CIs 0.76–0.79) with marked variations by gender (females 0.03, 95% CIs 0.00–0.06 lower than males), age (with ages 55 or more 0.08, 95% CIs 0.02–0.14 lower than 15–35 years) and number of chronic health conditions (3 or more conditions 0.14, 95% CIs 0.09–0.19 lower than those with 0 conditions). A pattern of response to HRQoL questions was also evident. Response was less likely among respondents speaking Aboriginal languages at home, living in non-urban settings, and experiencing multiple chronic health conditions.

**Conclusions:**

The SF-6D provides useful information on the HRQoL of Aboriginal South Australians. However, non-completion was pronounced among respondents speaking traditional languages and experiencing more chronic health conditions. Improved participation of vulnerable and health compromised respondents through culturally safe and relevant self-reporting HRQoL utility instruments is needed.

## Background

Marked improvements in mortality, continued increases in the prevalence of multiple chronic conditions [1, 2], and their influence on health related quality of life (HRQoL) are contributing to growing demands for healthcare and commensurately higher costs [2]. In the face of these challenges, improving health systems’ understanding of what health outcomes are produced among the people they serve, at what cost and for whom, is critical [3]. A similarly urgent need is for the knowledge developed to use appropriate metrics which reflect the perspectives of people at the centre of system activities, that is, patients and populations [4].

To meet these needs, patient reported outcomes are increasingly used for patient groups [4–7] and the broader populations to whom they belong. Patient level reports of HRQoL often make use of health utility measures which account for multiple HRQoL domains and produce a single, cardinal value describing a person’s health status at a particular time [8]. Their use at a population level provides context for aggregated patient reported outcomes at disease and service levels. They also assist with evaluating healthcare treatment and service programmes’ role in changing population health across time and within population sub-groups [9] for example, by facilitating group comparison by ethnicity, age and disease status.

Australia has a substantial history of using surveys to monitor population health status. In more recent times these have begun incorporating health utility measures nationally [10–12] and among state and territory jurisdictions. For example, South Australia’s long standing, annual Health Omnibus Survey (HOS) series is a random and representative household survey which has administered dedicated utility measures several times since their first inclusion in 1998 [13]. The SF-36 or its abridged form SF-12, is routinely included as a multi-dimensioned and generic HRQoL measure. To enable its wider use in assessing outcomes, SF-12 results were revised to yield the SF-6D health utility measure [14]. The SF-6D has subsequently been used to describe HRQoL norms for the Australian population [15].

Despite high quality, survey based data collections, the HRQoL of many specific population groups remains largely unknown [16]. For example, the disparities in health outcomes between Australia’s Aboriginal and Torres Strait Islander (herein respectfully referred to as Aboriginal) and non-Aboriginal populations is well documented in terms of: higher avoidable mortality and lower life expectancy; higher use of emergency and inpatient hospital services, particularly in areas of potentially preventable episodes of care; and the burden associated with chronic disease such as diabetes, cardiac and renal conditions.

Therefore, there is a need for supporting policy decisions and health system activities aimed at efficiently and equitably addressing peoples’ needs [4] by alleviating burden and improving HRQoL. However, the latter is not included in national frameworks tracking changes to Aboriginal health outcomes [17], nor is HRQoL and health utility of Aboriginal populations widely examined within jurisdictions. This is despite the fact that health utility and aggregated patient/population reported outcomes are increasingly used to inform decisions directly affecting Aboriginal Australians on issues ranging from selecting medications for subsidy [18] through to evaluating health service performance. Some exceptions are noted in Queensland where estimates for Aboriginal health workers [19] and Aboriginal cancer patients [20] are available. In South Australia, the SF-12 [21] has been used among remote Aboriginal South Australians living with diabetes [22]. However, it has not been validated among Aboriginal South Australians [21, 23] or used to report SF-6D health utilities [21]. Nor have the health preferences of Aboriginal Australians and their conceptions of health [21, 23] been contrasted against the outcomes of the generic SF-12 instrument. The use of existing generic HRQoL measures among Indigenous populations is a challenging area. Both national [24] and international [25] experience alerts us to characteristics associated with lower participation or impeded responding within health surveys. These characteristics include poor health literacy, illness severity, language barriers and cultural biases in the relevance of questions within instruments, and are more likely to affect Indigenous populations. These challenges are important to understand and respond to because non-participation is also associated with having relatively poorer health outcomes [4].

Employing the SF-12 among a representative population sample of Aboriginal South Australians would enable assessment of participation and question completion, provide a perspective on HRQoL, and facilitate comparison against wider South Australian and Australian population norms. The South Australian Aboriginal Health Survey (SAAHS) [26] provided an opportunity to pursue this. Having received funding through the Council of Australian Governments’ partnership on closing the gap in Aboriginal and non-Aboriginal health outcomes, SAAHS was commissioned to provide the first comprehensive estimates of chronic disease prevalence among Aboriginal South Australians.

This paper aims to conduct a descriptive analysis of HRQoL assessed within SAAHS using health utility as reported by Aboriginal South Australians using the SF-6D. In particular, we examine the characteristics of those completing HRQoL questions, the relationship between HRQoL and respondent characteristics, then position the HRQoL results in the wider South Australian and Australian population context.

## Methods

### Study design, setting and participants

The SAAHS [26] was a cross-sectional, face to face and representative survey of the Aboriginal population across metropolitan, rural and remote areas in the state of South Australia. SAAHS sampled from households within randomly selected Australian Bureaus of Statistics (ABS) 2006 Census collection districts using a stratified, multi-stage, clustered and self-weighted area design [27]. Participants were aged 15 years or more and identified as Aboriginal according to national best practice guidelines [28].

### Measurements

SAAHS administered 80 health related questions sourced from other population surveys, developed by the SAAHS Advisory committee, or previously validated instruments for population health assessment. The subset of questions available to our study included: socio-demographic characteristics of gender, age in 10-year groupings and urban, regional or remote area of residence; Aboriginal language use categorised as either English or Aboriginal/Aboriginal English as the main language at home; employment as under-employed, employed at home, or employed outside the home; yearly income as $20,000 or less. Interviewees were also asked whether a doctor had ever diagnosed them with any of the following conditions: diabetes; renal disease; hearing loss; mental health issues; asthma; or, hypertension. The number of chronic conditions reported by each respondent was summed and categorised as no conditions, 1 or 2 conditions, or 3 or more conditions.

#### Health utility outcomes

Health utility was estimated using the SF-6D [14, 29, 30] as based on the SF-12 version 2’s 12 items [31] and used under licence. The SF-6D version 2 uses six HRQoL subscales: physical function, role limitation, social function, bodily pain, mental health and vitality. The subscales combine for an overall utility score ranging from worst possible, or death equivalent, (0.39) to full HRQoL (1.00). UK general population utility weights derived by standard gamble techniques [14] were used in estimating Australian norms [15].

### Data analysis

Standard scoring algorithms were used to derive the SF-6D score for HRQoL. Where responses to SF-12 questions were missing (*n* = 61), no SF-6D score was recorded for that interview. The sample of SF-6D completed and scored (1) and not scored/missing (0) responses were compared on the basis of socio-demographic and health condition variables using logistic regression and we report the unadjusted odds ratios (OR) and their 95% confidence intervals (95%CIs). The distribution of completed SF-6D scores was negatively skewed. The distribution was improved using a cubic transformation, the results of which were used to affirm the adequacy of models reported herein. Interquartile range and arithmetic means for the untransformed scores are reported. So too are the results of ordinary least squares regression of SF-6D score against stratum within each available predictor. The reported beta coefficients and 95%CIs indicate the direction, size and strength of changing stratum levels on SF-6D score. These variables were trialled concurrently to derive the most parsimonious and best fitting model of SF-6D, the parameters of which were used to predict missing SF-6D scores. The predicted mean SF-6D scores for those originally completing/not completing SF-12 items were then compared using independent group t-tests. Age group results are contrasted against published age norms for Australia [15] and unpublished South Australian Health Omnibus [13, 32] results in 2008. All analyses were conducted with Stata version 15.1 [33].

## Results

A response rate of 57.7% saw 399 interviews completed from an initial sample of 691 eligible persons. Of those, 61 respondents completed demographic questions but not sufficient SF-12 items to enable scoring of the SF-6D. This group represented 10.9% (95% CIs 8.6–12.8) of Aboriginal South Australians aged 15 or more and Table 1 compares a selection of their characteristics with those who completed SF-6D health utility scores. On average, those completing SF-6D scores were less likely to: speak Aboriginal languages or Aboriginal English at home (OR = 0.32, 95% CIs 0.16–0.63); to live in regional or remote areas (OR = 0.12, 95% CIs 0.03–0.54 and OR = 0.01, 95% CIs 0.00–0.07 respectively); or experience at least one of the six chronic health conditions listed (1 or 2 conditions OR = 0.44, 95% CIs 0.23–0.86 and 3 or more conditions OR = 0.41, 95% CIs 0.19–0.91).

**Table 1:**
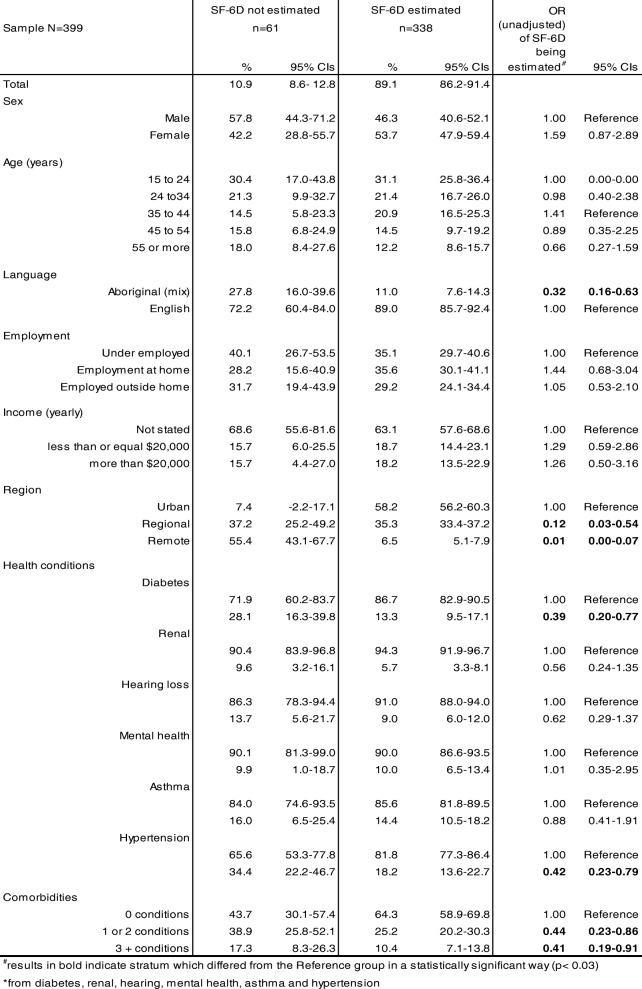
Respondent characteristics for completed health utility (SF-6D) estimation

Completed SF-6D scores ranged from 0.39 to 1.00 and were negatively skewed with a median of 0.82 and mean 0.77 (95% CIs 0.76–0.79) as shown in Table 2. On a bivariate level, mean scores varied across groups with females reporting lower health utility than males and age groups 35 years or more reported incrementally lower health utility compared to those aged 15 to 24 years. Speaking Aboriginal languages at home and living with chronic health conditions were also associated with lower health utility compared to those primarily speaking English at home and experiencing no chronic conditions respectively. Conversely, employment at home or outside the home was associated with comparatively better health utility than those who reported underemployment.

**Table 2:**
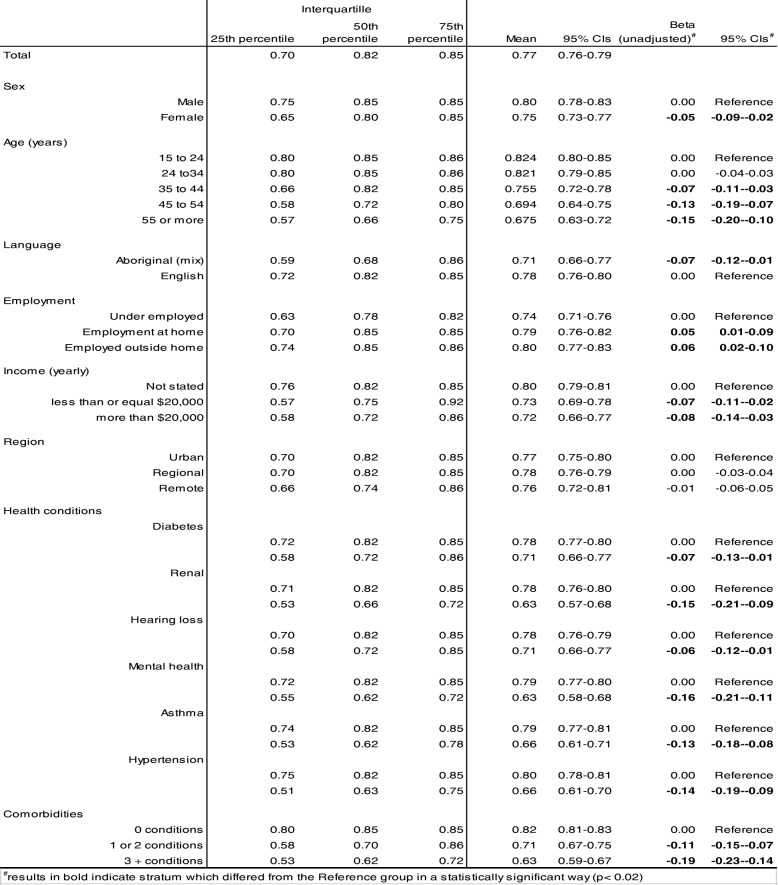
SF-6D by demographic and health conditions

These characteristics contributed to a multivariable model of health utility score (Table 3). The exception was language spoken at home which did not contribute significantly in the presence of other predictor variables. Concurrent assessment of each showed that females reported average health utility 0.03 (95% CIs 0.00–0.06) lower than males, age groups 35 years and beyond reporting incrementally lower health utility than those aged 15 to 34 years and living with chronic health conditions (1 or 2 conditions β = − 0.07, 95% CIs − 0.12- − 0.03 and 3 or more conditions β = − 0.14, 95% CIs − 0.19- -0.09). Employment continued to be associated with better health utility compared to underemployment by 0.05 (95% CIs 0.02–0.07). Overall, modelling gender, age, employment and chronic conditions as predictors of SF-6D scores explained 34.6% of the variance in those scores (r^2^ = 0.346, F(7,329) = 23.98, *p* < 0.001).

**Table 3:** Linear regression model of relationship between SF-6D scores and respondent characteristics

	Beta	95% Confidence Intervals		t	p	r^2^
		LCI	UCI			
Gender						
Male	0.00	Reference				
Female	-0.03	−0.06	−0.00	−2.22	0.027	
Age (years)						
15 to 34	0.00	Reference				
35 to 44	−0.05	−0.08	− 0.02	−3.32	0.001	
45 to 54	−0.09	−0.14	− 0.03	− 3.34	0.001	
55 +	−0.08	−0.14	− 0.02	−2.68	0.008	
Employment						
Under employed	0.00	Reference				
Employed	0.05	0.02	0.07	3.15	0.002	
Chronic health conditions						
0 conditions	0.00	Reference				
1 or 2 conditions	−0.07	−0.12	−0.03	−3.17	0.002	
3 + conditions	−0.14	−0.19	− 0.09	−5.18	< 0.001	
Constant	0.82	0.80	0.85	71.94	< 0.001	
Model fit (r^2^)						0.346

Using those parameters to predict SF-6D among missing responses resulted in that group having comparatively lower health utility at 0.75 (95% CIs 0.72–0.77), β = − 0.03 (95% CIs − 0.05- -0.00).

**Fig. 1:**
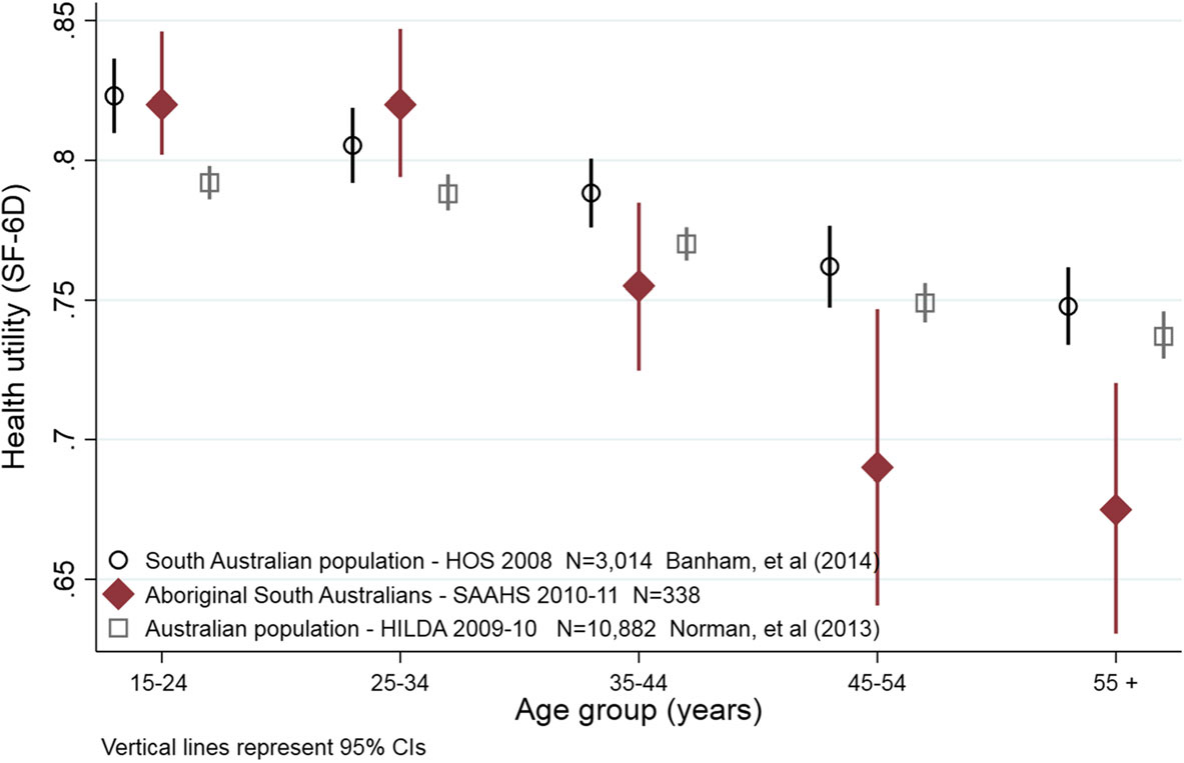
Population health utility (SF-6D) by age

Health utility among Aboriginal South Australians by age is placed in the wider South Australian (*n* = 3014) and Australian population (*N* = 17,630) context within Fig. 1. Health utility decreased across age groups for each of the three population groups. However, having observed very similar utility levels among those aged 15 to 34 years, the incremental decreases in health utility observed among Aboriginal South Australians in subsequent age groups was more pronounced than those in either of the comparator populations.

## Discussion

The rising prevalence of chronic disease and the risk of accumulating morbidity makes it increasingly important to monitor the HRQoL of populations. This is particularly so for groups already vulnerable to other forms of health loss through early death and the influence of widespread and pervasive social disadvantage, as is the case with Aboriginal Australians. This paper is one of few that describes HRQoL among Aboriginal Australians using a health utility measure. Having used a representative sample of randomly chosen Aboriginal adults [27] it provides a valuable comparator for reports of HRQoL within Aboriginal communities and across the broader community.

The mean HRQoL utility reported among Aboriginal South Australians aged 15 or more was 0.77 (95% CIs 0.76–0.79) which is equivalent to Australian norms of 0.77 (95% CIs 0.76–0.77) using data collected in 2009–2010 [15]. Underlying those average HRQoL levels were gender differences whereby Aboriginal females reported 5% lower HRQoL than males on average. This was consistent with the nature of gender differences observed in the wider Australian population using the SF-6D [15] and other health utility instruments such as the AQoL whether in South Australia [13] or nationally [11]. Declining HRQoL across age groups is also consistent with the general population norms [15]. However, two points of difference were notable. The average Aboriginal HRQoL at ages 15–24 was higher than that of the Australian population while the magnitude of health utility decrease into older ages within the Aboriginal community was markedly greater than those observed within the contemporary South Australian and Australian populations. This has important implications for interpreting mean health utility as each population has quite different age profiles. For example, as a consequence of high premature mortality rates, ages 45 and above are under-represented in the Aboriginal community in comparison to the non-Aboriginal population of South Australia and accounted for 17 and 41% of the respective populations [34]. Consequently, if Aboriginal South Australia had a similar age profile to that of the non-Aboriginal population, their average HRQoL utility would be lower by around 7%. Our results also provide clear evidence of pervasive self-reports of chronic health conditions and that multiple comorbid conditions are related to lower health utility. This is consistent with related population analyses. For example, the New South Wales’ 45 and up study demonstrated Aboriginal respondents were comparatively more likely to report poorer self-rated health and quality of life [35] and this was further exacerbated as the number of chronic conditions increased [35]. In addition, related follow-up studies of respondents by Aboriginality [36] found response rates were lower among those reporting poorer health status and lower quality of life [36].

This latter observation resonates with a further finding of interest in our analysis. While questions enabling the description of health utility were completed by most SAAHS interview respondents, a pattern of non-responding was also apparent. Some of the predictors of not completing health utility questions (language at home and chronic health conditions) were also indicators of poorer HRQoL. Using completed responses to predict HRQoL of those missing utility scores indicated significantly lower average health utility would be expected among the latter group (by approximately 2%).

In the context of a population already reporting lower HRQoL than the wider community when age profile is considered, our findings identified a further population sub-group whose perspective on HRQoL has not been given voice. Importantly, there is reason to believe this group has further reduced health utility. The relationship observed between poor health outcomes and language is also reported in other settings. For example, a study of Aboriginal cancer patients in Australia’s Northern Territory found those with an indigenous language experienced significantly poorer outcomes than those with English as their first language [37]. This suggested issues such as health literacy, depth of understanding of mainstream vernacular and difficulties in communicating within that paradigm may restrict the uptake of effective health care. The lack of engagement with SF-12 HRQoL questions may be similarly affected and the nature of questions considered too distant from, or irrelevant to, the circumstance of people whose traditional cultural connection remains strong [21, 23].

In its report to the South Australian Parliament, South Australia’s Health Performance Council [16] identified the opportunity for purposefully sampling specific populations to improve awareness of unmet health needs and encourage accountable responses by the health system. The SAAHS method [27] provided some evidence in support of using a standard, generic health utility measure as a means of meeting this information need for Aboriginal South Australians. Importantly though, the results suggest cautious use because a sub-group within the target population was identified as less likely to fully participate. Those less likely to self-report health utility were also more likely to have higher levels of comorbidity and experience poorer health utility.

This raises two limitations in our analysis. The first is to recognise a probable bias in our results whereby health utility among Aboriginal South Australians is over estimated because of the omission of a vulnerable population sub-group. Secondly, if language use contributes to the exclusion of people who can reasonably be considered as having ill-described and unmet HRQoL needs, then further research is required to remedy that with suitably adapted [24], culturally relevant [21, 22, 25] and validated [23] measures.

It is imperative to pursue these improvements and build on the strengths of this study which provided evidence of variations and disparate HRQoL utility among a representative sample of randomly selected Aboriginal adults [27]. These activities will help expand existing population health assessment beyond life expectancy, an acknowledged area of considerable inequity, to include informed discussion of a population’s perspective of their own HRQoL utility. Ultimately, HRQoL utility measurement could be subsumed into estimating healthy life expectancy [13, 38], a “best overall measure” p262 [39] and one widely reported internationally [40]. Healthy life expectancy helps reframe descriptions of population health disparity to include peoples’ experiences of morbid illness and its severity. Health utility measurement has a special role as it makes use of self-reported outcomes in a form salient to evaluating health system activities designed to address morbid illness and improve patient/population health outcomes [41].

## Conclusions

The SF-6D, as a generic health utility measure, provides useful information on the population health status of Aboriginal South Australians, albeit from a narrow and biomedically focussed perspective. However, caution is needed in its further use because the instrument’s questions were less likely to be responded to by people speaking traditional language, experiencing more chronic health conditions and reporting poorer health utility. Our results therefore suggest a need for improved instruments that are salient to the Aboriginal population and which lead to improved participation and self-reporting of HRQoL and health utility.

## Abbreviations

95% CIs: 95% confidence intervals
Aboriginal: Aboriginal and/or Torres Strait Islanders
ABS: Australian Bureaus of Statistics
HRQoL: health related quality of life
OR: Odds Ratio
SAAHS: South Australian Aboriginal Health Survey
